# Exploiting document graphs for inter sentence relation extraction

**DOI:** 10.1186/s13326-022-00267-3

**Published:** 2022-06-03

**Authors:** Hoang-Quynh Le, Duy-Cat Can, Nigel Collier

**Affiliations:** 1 Faculty of Information Technology, VNU University of Engineering and Technology, Hanoi, Vietnam; 2 Department of Theoretical and Applied Linguistics, University of Cambridge, Cambridge, UK

**Keywords:** Relation extraction, Graph, Deep learning, Convolutional neural network, Multiple paths

## Abstract

**Background:**

Most previous relation extraction (RE) studies have focused on intra sentence relations and have ignored relations that span sentences, i.e. inter sentence relations. Such relations connect entities at the document level rather than as relational facts in a single sentence. Extracting facts that are expressed across sentences leads to some challenges and requires different approaches than those usually applied in recent intra sentence relation extraction. Despite recent results, there are still limitations to be overcome.

**Results:**

We present a novel representation for a sequence of consecutive sentences, namely document subgraph, to extract inter sentence relations. Experiments on the BioCreative V Chemical-Disease Relation corpus demonstrate the advantages and robustness of our novel system to extract both intra- and inter sentence relations in biomedical literature abstracts. The experimental results are comparable to state-of-the-art approaches and show the potential by demonstrating the effectiveness of graphs, deep learning-based model, and other processing techniques. Experiments were also carried out to verify the rationality and impact of various additional information and model components.

**Conclusions:**

Our proposed graph-based representation helps to extract ∼50*%* of inter sentence relations and boosts the model performance on both precision and recall compared to the baseline model.

**Supplementary Information:**

The online version contains supplementary material available at (10.1186/s13326-022-00267-3).

## Background

Relation extraction (RE) is the task of discovering semantic connections between entities [1]. RE plays a vital intermediate step in a variety of natural language processing (NLP) and information extraction applications in the biomedical domain. Its applications range from precision medicine [2], adverse drug reactions identification [3, 4], drug abuse events extraction [5], major life events extraction [6, 7] to building question answering systems [8, 9] and clinical decision support system [10].

Most previous RE studies followed the assumption that if two entities were related, they would belong to a single sentence and therefore ignored relationships expressed across sentence boundaries [11–15]. I.e., the task of RE aims to classify the semantic relationship between an entity pair *e*_1_ and *e*_2_ in a given sentence *S* into a pre-defined relation class including ‘not-relate’. However, relationships between entities are often expressed across sentence boundaries or otherwise require a broader context to disambiguate [16–18]. For example, 30*%* of relations in the Biocreative V Chemical-Disease Relation (BC5 CDR) dataset [19] are only expressed across sentence boundaries, such as in the following excerpt expressing complicated inter sentence relations.

“<*Title*> Case report: acute unintentional **carbachol** intoxication.

…

< *S*_1_> **Carbachol** concentrations in serum and urine on day 1 and 2 of hospital admission were analysed by HPLC-mass spectrometry.

< *S*_2_> RESULTS: Minutes after oral administration, the patient developed ***nausea***, ***sweating*** and ***hypotension***, and finally collapsed.

< *S*_3_> ***Bradycardia***, cholinergic symptoms and ***asystole*** occurred. …”

*(PMID: 16740173)*

In which, chemical *‘carbachol’* is annotated to the Chemical-induced Disease (CID) relations with four diseases *‘nausea’*, *‘hypotension’*, *‘bradycardia’* and *‘asystole’*. All of them are inter sentence relations: *‘carbachol*’ only appears in the title and Sentence 1 while *‘nausea’* and *‘hypotension’* appear in Sentence 2 and *‘bradycardia’* and *‘asystole’* only appear in Sentence 3. These problems are exacerbated by the document- (rather than sentence-) level annotation, which is very common in the biological text [17].

Thus, the research community has gained an interest in devising methods to move beyond single sentences and extract semantic relations that span sentences. I.e., the task of inter sentence RE aims to identify the semantic relationship between a pair of entity mentions *e*_1_ and *e*_2_ in a given document *D* that contains several sentences *S*_1_,*S*_2_,...*S*_*n*_. The extraction of inter sentence relations is much more difficult than intra sentence relations [20]. In some datasets, the involved entities of an inter sentence relation are marked in specific locations (example includes BB3 corpus [21]). DocRed dataset [22] annotates the relations and entities together with their corresponding supporting sentences. The inter sentence relation extraction problem becomes much more difficult in the datasets that a relation explores entities at the document level rather than that at the specific mentions. I.e., since several mentions of an entity appear in different locations in the text, we face the difficulty in locating which sentences containing the supporting evidence of a relation. This problem becomes more severe in the biomedical domain since biomedical documents often contain sentences with a long and more complex structure compared with that in the general domain. Moreover, many relations are expressed implicitly. When working with multiple sentences, extracting valuable information, and then understanding the contexts of entity pairs becomes much more difficult. There is a multitude of different relation types in the biomedical domain and potentially any pair of entities in the document could be related. For example, although BC5 CDR corpus is only annotated with CID relations, many pairs of entities can have therapeutic relations.

These characteristics lead to some challenges and require different approaches than those usually applied in intra sentence relation extraction. Despite some initial results, there are still limitations of recent approaches for inter sentence RE. The end-to-end model proposed in [23] resolved intra sentence relation classification partly by using a multi-pass sieve coreference resolution module. It has the drawback of strongly depending on the appearances of antecedent and anaphor representations of entities in the text since there are many inter sentence relations not expressed through anaphor. Another approach processes consecutive sentences as longer sentences. Examples include a Support Vector Machine (SVM)-based model with a very rich feature set [24], a hybrid model of the convolutional neural network, and maximum entropy (ME) [25] and a long short-term memory network (LSTM) and convolutional neural network model that learns document-level semantic representations [20]. Since inter sentence RE requires information from all local, non-local, syntactic, and semantic dependencies, several previous studies tried to build a representation for the whole document such as biaffine Relation Attention Networks (BRANs) [17] and the labeled edge graph convolutional neural network model on a document-level graph [18].

The novel approach we present in this paper draws inspiration from related works that explore the consecutive sentences for the inter sentence relation extraction. The construction of document subgraphs is also used to leverage both local and non-local information effectively. We then construct a deep neural architecture based on a shared-weight convolutional neural network (swCNN) with an improved attention mechanism to explore the information of multiple paths on the document subgraph. The experimental results on the BC5 CDR benchmark dataset show potential and are comparable to state-of-the-art approaches. The investigation of the impact of different components and information on the final performance provides insights showing that the graph-based representation, swCNN model, instance merging/weighting technique and distant supervision learning are useful. It also leads us to conclude that the knowledge-based information, coreference information and attention mechanism are still promising areas for future research.

## Materials and methods

We present this section in four main parts: the overview of our evaluated dataset; the overall picture of the proposed architecture and three main components in detail; additional techniques to improve model performance; and experimental configuration.

### Dataset

Our experiments were conducted on the BioCreative V Chemical-Disease Relation dataset [19]. This corpus contained a total of 1500 PubMed articles that were separated into three subsets, each of 500 for the training, development and test set (the details are shown in Table 1). This dataset is annotated with chemicals, diseases and the chemical-induced disease relationships at abstract-level. Relation annotations are asserted for both within and across sentence boundaries. Following the data survey of BioCreative [26], about 30% of total instances are inter sentence relationships.

**Table 1:** Summary of the BioCreative V CDR dataset

Subset	Abs	Disease			Chemical			CID
		Ment	ID	IAA	Ment	ID	IAA	
Training	500	4182	1965	0.8600	5203	1467	0.9523	1038
Development	500	4244	1865	0.8742	5347	1507	0.9577	1012
Test	500	4424	1988	0.8875	5385	1435	0.9630	1066

*Abs* Abstracts, *Ment* Mentions, *CID* Chemical-induced disease relations

### Model overview

**Fig. 1:**
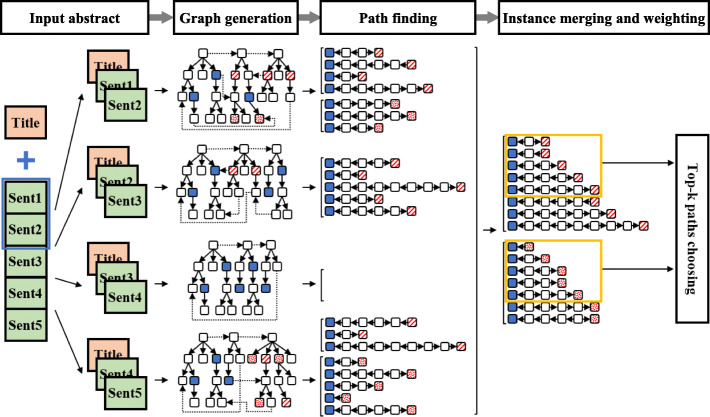
Proposed model for inter sentence relation classification. Red dotted and striped nodes indicate two types of disease. Blue filled nodes indicate one type of chemical

Figure 1 illustrates our proposed model for extracting the semantic relation at the abstract level, which contain four main phases: (*i*) Firstly, we construct a document subgraph to represent the relationship between entity pairs. (*ii*) In order to represent an instance by a set of paths, we apply several advanced techniques for finding, merging and choosing the relevant paths between entity pairs. (*iii*) In the next step, the advanced attention mechanism and several types of linguistic information are applied to explore the information from the document subgraphs more effectively. (*iv*) Lastly, to exploit these enriched representations effectively, we develop a shared weight Convolutional Neural Network model (swCNN).

### Document subgraph construction

As we noted above two entities that participate in a relation may belong to different sentences. Dependency trees are often used to extract local dependencies of semantic relations in intra sentence relation extraction. However, such dependencies are not adequate for inter sentence RE since sentences have different dependency trees that are not connected. Because of this limitation, using the shortest dependency path to extract the local dependencies of semantic relations is not adequate for inter sentence RE.

To overcome these limitations, we construct a graph for consecutive sentences based on their dependency trees, called the ***document subgraph***. In this graph, the nodes correspond to words and edges represent the connection between them. We make two assumptions: *(i)* the distance of two participating entities in a relation should not be too far (experimentally, two entities should be within five consecutive sentences). If two entities are too far apart, the method’s effectiveness would be reduced, or this pair may be ignored. *(ii)* The title of the abstract is a special sentence that is related to every sentence in the abstract in a certain manner. Because of this assumption, the title is always used together with the abstract sentences to generate each subgraph.

Creating a document subgraph is a three-step process:

***Step 1:*** Generate the dependency tree for each sentence. All directed dependency labels are kept in the subgraphs as local dependency information.

***Step 2:*** Merge the dependency trees of the sentences in each sliding window into a document subgraph.

The sliding window of size *w* indicates the number of consecutive sentences that we use to create the document subgraphs. *w*=1 indicates a single sentence, i.e. the model only extracts the intra sentence relations. With *w*=*j*, each *j* sentences are used to create a subgraph. Since two entity mentions can appear in different sentences, an unrestricted selection of text spans would risk generating many unexpected examples and lead to an explosion of computing space (see Instance merging.) We, therefore, limit *w* to 5, i.e., all relations with two entities that are not within 5 consecutive sentences are ignored. After this phase, each abstract will consist of several subgraphs.

***Step 3:*** Create virtual edges for subgraphs. By using dependency trees, we already have local dependency information. In this step, we try to link new virtual edges by using several additional information:

- **NEXT-SENT** edges connect root nodes in dependency trees of two consecutive sentences. They bring sequential non-local dependency information.
- **TITLE** edges are created between two dependency tree roots of the Title and the first sentence in the sliding window. They provide non-local dependency information.
- **COREFERENCE** edges link an anaphoric expression to its antecedent if identified by the multi-pass sieves coreference resolution method [23]. These edges show the semantic relation between terms. We divide this connection type into three specific types: (i) COREF-sent: anaphor and antecedent belong to two normal sentences, (ii) COREF-to-title: anaphor is in a normal sentence and antecedent is in the Title, (iii) COREF-from-title: anaphor is in the Title and antecedent is in a normal sentence.
- **KB-CTD** edges are created between head nodes of two entities if they are annotated as having relation *‘M’* in the Comparative Toxicogenomics Database (CTD)^1^. We call it knowledge-based information.

These virtual edges are *undirected* and labeled by their names. We give a realistic example of the document subgraph in Additional file 1: Appendix A.

Using the subgraphs already constructed, this module finds all possible paths between two entities in each graph. We perform a breadth-first search on a graph to find all possible paths between two entities. The graph we constructed is quite complex, moreover, the complexity increases with the sliding window size *w* and the number of new virtual edges. A traversal in breadth-first order on such a large graph with cycles is resource-consuming (even if we never go back to the passed nodes to avoid the infinite issue).

To overcome this risk, we use two *thresholds*:

- Maximum depth *md*: The maximum number of nodes traveling from the beginning node.
- the Maximum number of path *k*: The maximum number of paths that we collect.

Nearly all previous studies in relation extraction consider co-occurring entity pairs with known relations as positive instances for training. This assumption is reasonable for intra sentence relations, but the inter sentence problem presents a new challenge since this strategy would risk generating too many wrong examples. It is because a document has a relation between two entities does not mean that all spans of text contain these entities show that relation. Quirk and Poon [16] tackled this problem when an entity pair co-occurs in a large text span, and also co-occur in a smaller text span that overlaps with the larger one. In such cases, if there is a relation between the pair, most likely it is expressed in the smaller text span when the entities are closer to each other. To reduce the unexpected noise from the large text span, we apply a restriction of generating paths called *‘minimal span’* [16]. I.e., only the minimal span is chosen to generate the paths between two entities. A co-occurring entity pair has the minimal span if there does not exist another overlapping co-occurrence of the same pair. Since each abstract can have several subgraphs, in this phase, we receive several sets of paths.

#### Instance merging

**Fig. 2:**
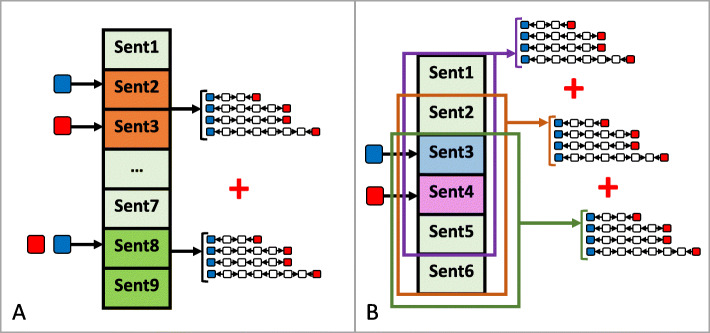
Examples of two unexpected problems while generating the instance from document subgraph

Figure 2 illustrates the instance merging technique. Firstly, we address two unexpected problems while generating the instance from the document subgraph. In Fig. 2-A, a pair of entities appear several times at different positions in an abstract. Because the BC5 CDR corpus has relations annotated at the abstract-level, all of these co-occurrences are treated as positive examples for the CID relation. In fact, only a few of them actually refer to the CID relation. This may cause much noise during training.

The example in Fig. 2-B shows the problem of unexpected instance repetition, especially when we widen the window to create subgraphs. In this example, we can generate three identical training instances, i.e., the training patterns of this instance are produced three times, changing the actual frequency of the representation in the training data. This issue may then lead the model to give this instance a higher priority (more important weight). We give a realistic example of these problems below:

“<*Title*> ***Hemolysis*** of human erythrocytes induced by **tamoxifen** is related to disruption of membrane structure.

…

< *S*_1_> **TAM** induces ***hemolysis*** of erythrocytes as a function of concentration.

< *S*_2_> The extension of ***hemolysis*** is variable with erythrocyte samples, but 12.5 microM **TAM** induces total ***hemolysis*** of all tested suspensions.

< *S*_3_> Despite inducing extensive erythrocyte lysis, **TAM** does not shift the osmotic fragility curves of erythrocytes.

< *S*_4_> The hemolytic effect of **TAM** is prevented by low concentrations of alpha-tocopherol (alpha-T) and alpha-tocopherol acetate (alpha-TAc) (inactivated functional hydroxyl) indicating that **TAM**-induced ***hemolysis*** is not related to oxidative membrane damage.

< *S*_5_> This was further evidenced by absence of oxygen consumption and hemoglobin oxidation both determined in parallel with **TAM**-induced ***hemolysis***. …”

*(PMID: 10704919)*

Tackled with a title and 5 sentences as shown above and a sliding window size *w*=3, we have 42 valid pairs of CID: TAM-Hemolysis. Each entity pair can potentially be described by up to 15 paths. As a result, if each pair CID: TAM-Hemolysis is considered as a positive instance, we may have too many ‘similar’ positive instances. The same problem also appears for negative instances. To solve this problem, we propose a technique called ***instance merging***, in which, we extract all possible dependency paths between a pair of entity mentions and merge them into a single set for this entity pair. To reduce overlapping training instances, we remove the repeated paths (i.e., if several paths are totally identical, only one is kept).

#### Choosing top −*k* paths

After the instance merging phase, we have a set of several paths to represent a pair of entities. Some of them are useful, but others may be noise.

Prior works on intra sentence relation extraction often explored the single shortest path between two entities [27, 28]. Applying these traditional approaches for inter sentence relation classification problem raises many problems. Firstly, we cannot take advantage of all the local and global features since they may appear in different paths; secondly, the shortest path may not the ‘best’ path.

In contrast to these previous approaches, we propose to consider a ***set of multiple paths*** as a novel representation for an entity pair. To reduce noise and model complexity, we only choose the top-*k* best paths. This leads to the problem of how to choose advantageous paths. In this work, we implement two strategies to choose the top-*k* paths:

- Top-*k* shortest dependency paths, this strategy was also used by [16].
- Top-*k* paths with the highest number of repetitions.

To explore the information in this novel representation, we cannot use our previous models. Instead, a new deep learning architecture capable of simultaneously processing multiple paths was proposed, based on the swCNN.

### Path representation

Before inputting to the model, each component in the dependency paths must be transformed into an embedding vector. In order to have an informative representation, we take advantage of various linguistic information along the dependency path, from the original dependency tree and other resources.

The ***dependency relations*** with directions are proven more effective than the dependency relations without directions for the relation extraction task [27]. However, treating the dependency relations with the opposite direction as two separate relations can induce two vectors for the same relation. We represent the dependency relations with two discrete components: ${\mathbf{d}}^{typ}\in {\mathbb{R}}^{\dim_{typ}}$ represents the dependency relation type among 72 labels; and ${\mathbf{d}}^{dir}\in {\mathbb{R}}^{\dim_{dir}}$ is the direction of the dependency relation, i.e. from left-to-right or vice versa on the Shortest Dependency Path (SDP). The final representation **d**_*i*_ of dependency relation is obtained through a nonlinear transformation as follow:

1 $$ \mathbf{d}_{i} = \tanh\left(\left[ \mathbf{d}^{typ}_{i} \oplus \mathbf{d}^{dir}_{i} \right] \mathbf{W}_{d} + \mathbf{b}_{d} \right)  $$

where the **d**^*typ*^ and **d**^*dir*^ vectors are generated by looking up the embedding matrices ${\mathbf{W}}_{typ}^e\in {\mathbb{R}}^{\dim_{typ}\times 72}$ and ${\mathbf{W}}_{dir}^e\in {\mathbb{R}}^{\dim_{dir}\times 2}$ respectively; **W**_*d*_ and **b**_*d*_ are trainable parameters of the network.

For token representation, we utilize two types of embeddings to represent the word information in different aspects, including:

- *Pre-trained fastText embeddings* [29] learn the word representation based on its external context and *n*-gram sub-word information. Each token in the input paths is transformed into a vector $\mathbf {t}^{w}_{i}$ by looking up the embedding matrix $$ {\mathbf{W}}_w^e\in {\mathbb{R}}^{\dim_{we}\times \left|V\right|} $$, where dim_*we*_ is the word embedding dimension, and *V* is the vocabulary of all words we consider.
- *POS tag embeddings* captures (dis)similarities between grammatical properties of words and their syntactic structural roles within a sentence. We concatenate the part-of-speech (POS) tag information into the token representation vector. We randomly initialize the embeddings matrix $$ {\mathbf{W}}_p^e\in {\mathbb{R}}^{\dim_{pe}\times 56} $$ for 56 OntoNotes 5.0 version of the Penn Treebank POS tags. Each POS tag label is then represented as a corresponding vector $\mathbf {t}^{p}_{i}$.

We concatenate two embedding vectors of each token and transform them into the final token embedding as follow:

2 $$ \mathbf{t}_{i}=\tanh\left(\left[\mathbf{t}^{w}_{i}\oplus\mathbf{t}^{p}_{i}\right]\mathbf{W}^{t}+\mathbf{b}^{t} \right)  $$

Each token **t**_*i*_ is concatenated with the corresponding **attentive augmented information** from its child nodes on the original dependency tree proposed by Can et al. [30]. Given a token *t*, the attentive augmented information is calculated using the token itself and the set of its *M* child nodes. Word embedding and POS tag embedding are concatenated to form token embedding vector **t** while the dependency relation from a direct ancestor is added to form a child node representation **c**_*i*_. The position embeddings *d*_*i*_ is also used to reflect the relative distance from the *i*-th child to its parent on the original sentence.

Two sequential attention layers on the children of a token are used to produce children context vectors. A simple self-attentive network is applied to child nodes $\left \{ \mathbf {c_{i}} \right \}^{M}_{i=1}$ where the attention weights are calculated based on the concatenation of themselves with parent information and distance from the parent. I.e.,

3 $$ \begin{aligned} \bar{\mathbf{C}} &= \left\{ \mathbf{c}_{i} \oplus \mathbf{t} \oplus d_{i} \mathbf{w}_{d} \right\}^{M}_{i=1} = \left\{\bar{\mathbf{c}}_{i}\right\}^{M}_{i=1}\\ \mathbf{e}&=\left\{ \bar{\mathbf{c}}_{i} \mathbf{W}_{e} + b_{e} \right\}^{M}_{i=1}= \left\{ e_{i} \right\}^{M}_{i=1}\\ \alpha^{s}_{i}&=\text{sigmoid}(e_{i})\\ \mathbf{c}^{s}_{i}&=\alpha^{s}_{i} \mathbf{c}_{i} \end{aligned}  $$

where ${\mathbf{w}}_d\in {\mathbb{R}}^{\dim_d}$ is the base distance embedding; **W**_*e*_ and *b*_*e*_ are weight and bias term.

A distance-based heuristic attentive layer is applied on the self-attentive children context vector to keep track of how close each child is to the target token, as follow:

4 $$ \begin{aligned} \alpha^{h}_{i}&=\text{sigmoid}(\beta d^{2}_{i})\\ \mathbf{c}^{h}_{i}&=\alpha^{h}_{i} \mathbf{c}^{s}_{i} \end{aligned}  $$

where *f*(*d*)=*βd*^2^ with *β*=−0.03 is a heuristically chosen weighting function.

Afterward, to capture the relevant and essential information from the output of the multi-attention layer and preserve the integrity of the word information, *K* kernel filters are applied to each child’s attentive vector to produce *K* features from each child. The final augmented information **a** is captured by a max-pooling layer, i.e.,

5 $$ \begin{aligned} \mathbf{F}&= \left\{ \text{ReLU}\left(\mathbf{c}^{h}_{i} \mathbf{W}_{f} + \mathbf{b}_{f} \right) \right\}^{M}_{i=1}\\ \mathbf{a} &= \left\{ \max \left(\mathbf{F}^{\intercal}_{k} \right) \right\}^{K}_{k=1} \end{aligned}  $$

where **W**_*f*_ is the weight of *K* kernel filters; and **b**_*f*_ is bias term.

Finally, this concatenation is transformed into an *X*-dimensional vector to form the representation ${\mathbf{x}}_i\in {\mathbb{R}}^X$ of the token, i.e.,

6 $$ \mathbf{x}_{i} = \tanh\left(\left[ \mathbf{t}_{i} \oplus \mathbf{a}_{i} \right] \mathbf{W}_{x} + \mathbf{b}_{x} \right)  $$

where **W**_*x*_ and **b**_*x*_ are trainable parameters of the network.

### Shared-weight convolutional neural network

Convolutional Neural Networks (CNNs) [31] are good at capturing the *n*-gram features in the flat structure and have also been proved effective in many natural language processing tasks including relation classification [14, 17]. The typical structure of a shared-weight CNN (swCNN) is quite similar to the original CNN that is comprising convolution, pooling, fully-connected layers and softmax. The novel point is the ability to share weight between several convolutions, leading to the ability to process multiple data instances at once.

**Fig. 3:**
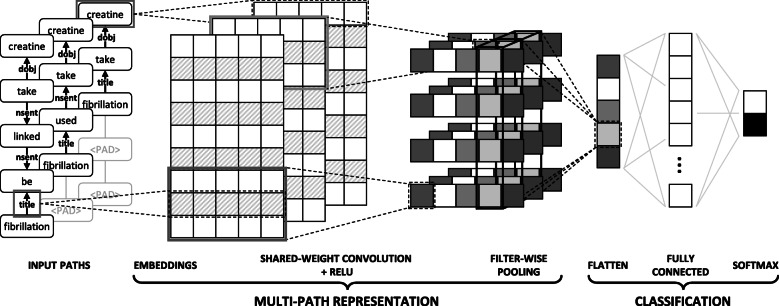
Diagram illustrating of a swCNN architecture

Figure 3 illustrates the overall architecture of our swCNN model, which is comprised of two main components: multi-path representation and classification. Given a set of multiple *k* paths as input, each path is converted into a separated embedding matrix. A shared-weight convolution with relu activation layer is followed to capture convolved features from these embedding matrices simultaneously. The essential features are gathered using a filter-wise pooling layer before being classified by a fully connected layer with softmax classification.

In the **embeddings layer**, each component in the dependency path (i.e., token or dependency relation) is represented by a *d*-dimensional vector ${\mathbf{w}}_e\in {\mathbb{R}}^d$ where *d* is the desired number of embedding dimensions as described in the previous section ‘Path representation’.

After the embeddings layer, the input multiple paths are transformed into:

7 $$ \mathbf{mP} = \left[\mathbf{x}_{i,1}, \mathbf{d}_{i,1}, \mathbf{x}_{i,2},...,\mathbf{x}_{i,n-1}, \mathbf{d}_{i,n-1}, \mathbf{x}_{i,n} \right]_{i=1}^{k}  $$

In general, let us define the vector **x**_*i*,*j*:*j*+*m*_ as the concatenation of *m* tokens and *m*−1 dependency relation between them. I.e.,

8 $$ {}\mathbf{x}_{i,j:j+m} = \mathbf{x}_{i,j} \oplus \mathbf{d}_{i,j} \oplus \mathbf{x}_{i,j+1} \oplus... \oplus \mathbf{d}_{i,j+m-2} \oplus \mathbf{x}_{i,j+m-1}  $$

In the **convolution layer**, we apply *N* filters with region size *r* to these embedding matrices simultaneously. These filters move by dependency unit to keep the dependency information between tokens. Since the same filters are used for all matrices, our model can extract information from them at the same time, as well as suppress increases in the number of weight parameters then reduce the computational complexity. The filter-wise pooling step converges all outputs of a filter to a single element by choosing the essential feature from all CNN features. This architecture helps swCNN to use the information on multiple paths simultaneously, and from there, selects the truly outstanding features. I.e., the convolutional layer computes an element **f**_*p*_ of the convolved feature vector **f** as follows:

9 $$ \mathbf{f}_{p} = \max_{\substack{1 \leq i \leq k \\ 0 \leq j \leq n-r+1}} \left[ \mathbf{x}_{i,j:j+r} \mathbf{W}_{c} + \mathbf{b}_{c} \right]_{p}  $$

where ${\mathbf{W}}_c\in {\mathbb{R}}^{\left( rX+\left(r-1\right)D\right)\times N}$ and ${\mathbf{b}}_c\in {\mathbb{R}}^k$ are the weight matrix and bias vector of the convolutional layer.

At the **classification** phase, we have the number of features equal to the number of filters we used. They then are flattened into a feature vector and put through the softmax to decide the final prediction. I.e., the output **f** of the convolutional layer is then fed to a softmax classifier to predict a (*K*+1)-class distribution over labels $\hat {\mathbf {y}}$:

10 $$ \hat{\mathbf{y}} = \text{softmax} \left(\mathbf{f} \mathbf{W}_{y} + \mathbf{b}_{y} \right)  $$

where **W**_*y*_ and **b**_*y*_ are the parameters of the network to be learned.

The proposed model can be stated as a parameter tuple *θ*=(**W**,**b**). To compute the model parameters *θ*, we define the training objective for a data sample as:

11 $$ L(\theta) = -\sum_{i=0}^{K} \mathbf{y}_{i} \log \hat{\mathbf{y}}_{i} + \lambda \left\| \theta \right\|^{2}  $$

where **y**∈{0,1}^(*K*+1)^ indicates the one-hot vector represented ground truth; and *λ* is a regularization coefficient.

### Additional techniques

#### Ensemble mechanism

Overfitting is one of the most notable problems of deep learning models. It happens when the neural network is very good at learning its training set, but cannot generalize beyond the training set (known as the generalization problem). The ensemble method [32] is one of the most effective paradigms to reduce variance and helps to avoid overfitting as well as improve the stability and accuracy of the model. Moreover, random initialization is demonstrated to have an impact on the model’s performance on unseen data, i.e. training model instances may perform substantially better (or worse) than the averaged results [17, 28, 33]. An ensemble mechanism was found to reduce variability whilst yielding better performance than the averaging mechanism [17].

In this paper, we use a strict majority vote – a simple but effective ensemble method that has been successfully used in some related works [28, 33]. Our ensemble system runs the model 20 times and uses the strict majority vote to obtain the final results.

#### Distant supervision learning

Distant supervision learning is proved its good impact on the relation classification by utilizing the knowledge base in some research [17, 23, 24]. In this work, we continue to apply distant supervision learning to the proposed subgraph models.

In order to take advantage of the available resources, we do not rebuild the distant data ourselves. Instead, we use the CTD-Pfizer dataset [34] that has been successfully applied in [17, 24]. Since this data does not contain entity annotations, we used Dnorm [35] and tmChem [36] tools to annotate the entities. This dataset contains 18,410 documents with 33,224 CID pairs (15,439 unique).

### Experimental configuration and model’s hyper parameters

Our model was implemented using Python version 3.5 and TensorFlow v1.15.0^2^. The dependency tree is generated using spaCy^3^. To generate the document subgraph, we set the maximum depth of *md*=15 and the maximum number of paths *k*=150 for the breadth-first search algorithm of pathfinding phase. Widening *w* more than 5 as it may bring a lot of noise information and cause a computational burden. Therefore, we limit the size of the sliding window *w* lower than 5, i.e., exclude all entity pairs that are apart more than 5 consecutive sentences. Heuristically, we choose top-*k* path with *k*=3 for each entity pair.

The shared weight CNN employs the *Adam* optimizer [37] and uses *Glorot* random uniform [38] initialization. The mini-batch training size is set to 128. Surveying the data has shown an undesirable consequence of the subgraph representation. That is an unexpected increase in negative data. For intra sentence problem, the ratio of positive and negative is about 1:2. But using the subgraph this ratio is 1:2.95, 1:3.53, 1:3.85, 1:4.05 and 1:4.20 respectively for window sizes 1, 2, 3, 4 and 5 (note that the title is always connected to the first sentence in sliding window). This leads to an imbalanced data problem, which may negatively influence system performance caused by the bias to the negative label. To minimize the impact of this problem, we assign the class weights to give priority to the minor classes (positive). At this time, we cannot learn this weight automatically. Therefore, we set them heuristically as 3:1 for *positive*:*negative*.

We fine-tuned our deep learning model using training and development subsets (as described in Table 1). The optimized model’s hyper-parameters in detail are shown in Table 2. For the final results, we use these configurations to run the training process 100 times and report the average results of 100 runs. The training time for each run is about 17.5 hours. The prediction time for the BC5 test set using the trained model is about 2 minutes.

**Table 2:** Tuned hyper-parameter of the proposed model

Information		Configuration	Parameters
Dependency embeddings	Dependency type	LUT $\mathbf {W}^{e}_{typ}$ size 72×150	10800
	Dependency direction	LUT $\mathbf {W}^{e}_{dir}$ size 2×150	300
Token embeddings	FastText embeds	Pre-trained 300−dim vector	−
	Character embeddings	LUT $\mathbf {W}^{e}_{c}$ size 85×50	4250
		biLSTM with 50 units	40400
	POS tag	LUT $\mathbf {W}^{e}_{t}$ size 57×50	2850
	WordNet embeds	Fixed spare 45−dim vector	−
Augmented information	Base distance embeds	32−dim vector	32
	Self attention score	**W**^*e*^,**b**^*e*^ transform from 832 dim to scalar	833
	Heuristic attention	Linear	−
	Kernel filters	100 filters size 832×1	83300
Shared weight-CNN		128 filters each region-size (1,2,3)	2056320
Classifier	Fully-connected MLP	Do not use	−
	Softmax	2 classes	768
Total number of parameters			2199853

Embed: Embedding, Dim: Dimension

We also apply some techniques to overcome overfitting, including *max-norm regularization* for Gradient descent [11]; adding *Gaussian noise* [13] with the mean of 0.001 to the input embeddings; applying *dropout* [39] at 0.5 after all embedding layers and CNN layers; and using *early stopping* technique [40].

## Results

We present this section in four main parts: the contribution of proposed virtual edges; the effectiveness of subgraph windows sizes, the ablation test results of the model components; and the comparison between our results and other state-of-the-art models.

### Effect of the injected virtual edges in the document subgraph

**Fig. 4:**
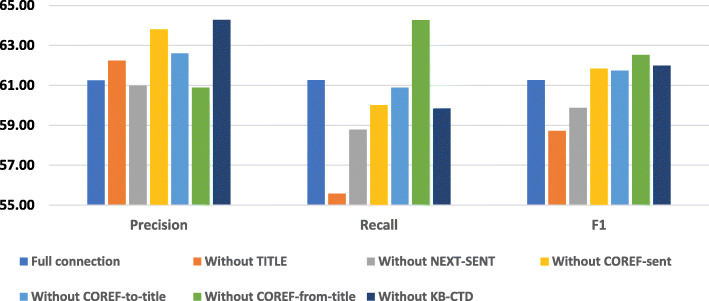
Ablation test results for virtual edges of the document subgraph. The vertical axis shows the performance in %. Experiments are conducted with 3 shortest paths

We study the contribution of injecting virtual edges on the system performance by ablating each of them in turn from the graph and afterward evaluating the model with the sliding window size *w*=2 and top-3 shortest paths for each entity pair (*k*=3). We compare these experimental results by the changes of Precision (*P*), Recall (*R*) and *F*1-measure in Table 3 and Fig. 4.

**Table 3:** Ablation test results for added virtual edges in the document subgraph

	Precision	Recall	F1
**Full connection**	*61.25*	*61.26*	*61.25*
Without TITLE	62.24	**55.58**	**58.72**
Without NEXT-SENT	**60.98**	**58.79**	**59.86**
Without COREF-sent	63.80	**60.01**	61.85
Without COREF-to-title	62.60	**60.89**	61.73
Without COREF-from-title	**60.88**	64.27	62.53
Without KB-CTD	64.28	**59.84**	61.98

Results are reported in %

Decreased results are highlighted in bold

This experiment presents an exciting view of the contributions for each type of virtual edge in the document subgraph. When removing NEXT-SENT from the graph, the results decrease in terms of all *Precision*, *Recall* and *F*1. The same results appear when we remove TITLE.

In addition, although the COREF-sent, COREF-to-title and KB-CTD help to find some more correct relations, it brings too many false-positive results and leads to worse *Precision* (removing them boosts the *Precision* but gives a bit lower *Recall*).

Using the COREF-from-title connection also reduce *F*1, but because it adversely affects heavily *Recall* whist only gives a minimal contribution to *Precision*.

These experimental results have raised a challenge that if we want to use the information about coreference and knowledge-bases, we need some additional methods to increase the quality of the information obtained. We left this problem for further work. Therefore, in the next experiments, we only use two connections NEXT-SENT and TITLE.

### Effect of different sliding window size *w* for training and testing

**Fig. 5:**
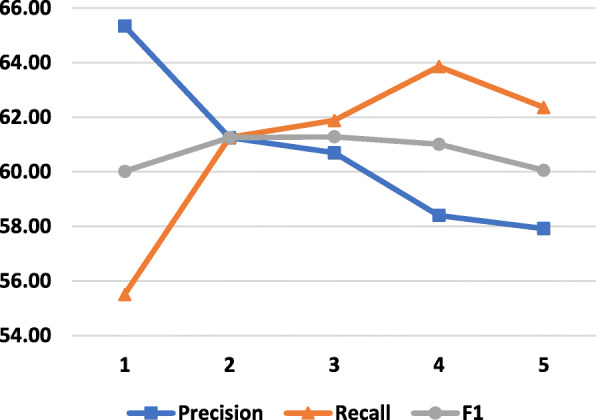
The change of results with different size of sliding window. The vertical axis shows the performance in % while the horizontal axis shows the size of *w*. Only fastText word embedding is used to represent words. Experiments are conducted with 3 shortest paths

We describe the change of the model’s performance with different sizes of the sliding window in Fig. 5. The larger *w* helps to increase *Recall* but leads to a worse *Precision*. This is an easy-to-explain result because with a larger *w* we will get more paths, but more noise. The equilibrium point of *Precision* and *Recall* gives the highest *F*1 result at *w*=2, in detail, we have *Precision*=61.25*%*, *Recall*=61.26*%* and *F*1=61.25*%*.

More importantly, this statement also raises an idea to take advantage of a large *w* but minimize the impact on *Precision* at the lowest level that whether we use the different window sizes for training and testing. The larger window size for training helps to collect new patterns in the text. The smaller window size for testing helps to reduce noise and narrow the allowed distance between two entities. To demonstrate this idea, grid search experiments with *k*=3 were conducted, the results are shown in Table 4.

**Table 4:** Results of the document subgraph with different sizes of the sliding window for training and testing

*w* for training	*w* for testing	Precision	Recall	F1
1	1	**65.34**	55.50	60.02
	2	62.20	57.22	59.61
	3	61.47	58.27	59.83
	4	61.92	54.86	58.18
	5	57.13	59.76	58.42
2	1	61.95	60.19	61.06
	2	61.25	61.26	61.25
	3	61.97	60.30	61.12
	4	61.30	58.52	59.88
	5	60.99	59.36	60.16
3	1	61.05	61.74	61.39
	2	60.65	61.74	61.19
	3	60.70	61.88	61.28
	4	62.30	59.47	60.85
	5	61.10	59.81	60.45
4	1	60.30	64.01	62.10
	2	57.88	**65.98**	61.67
	3	58.31	65.27	61.59
	4	58.40	63.86	61.01
	5	59.97	61.71	60.83
5	1	61.15	63.76	62.43
	2	60.13	65.89	**62.88**
	3	58.56	65.79	61.96
	4	58.64	62.42	60.47
	5	57.92	62.36	60.06

Results are reported in %

The highest result in each column is highlighted in bold

The results have verified the effectiveness of the proposed ideas. With the larger *w* for training size, we have better *Recall* but worse *Precision*. For each training window size, the smaller *w* for testing always brings better *F*1 than the larger *w*. The best *F*1 archived with *w*=5 for training and *w*=2 for testing, increase 1.34*%* compared to the best results of using the same window size for training and testing.

### Contribution of the model components

We further investigate the contribution of each component in Table 5, which shows changes in *F*1 when ablating each component from the proposed model.

**Table 5:** Ablation test results for various components of the document subgraph based model

Component removed/changed	Precision	Recall	F1	Change of F1
**Full model**	**60.13**	**65.89**	**62.88**	
Without subgraph	57.68	55.16	56.39	-6.49
Without TITLE	61.12	54.12	57.41	-5.47
Without NEXT-SENT	62.36	58.33	60.28	-2.60
Without instance merging technique	52.40	69.26	59.66	-3.22
Without swCNN and top-*k* paths	59.92	62.19	61.03	-1.84
Choose top-*k* by highest frequency (instead of length)	58.56	66.96	62.48	-0.40
Use w=2 for both training and testing (instead of different *w*)	61.25	61.26	61.25	-1.62
Without using class weight	59.60	65.92	62.60	-0.28
Without attention mechanism	59.13	64.85	61.86	-1.02

Results are reported in %

Column ‘Change of F1’ shows the decrease of F1 when removing/changing components from the model

Highest result in each column is highlighted in bold

The *F*1 reductions illustrate the contributions of all proposals to the final result. However, the level of contribution is varied among the different components. The document subgraph has proven its superiority by boosting the *F*1 by 6.49*%*, in which the *Recall* increases 10.73*%*. Both TITLE and NEXT-SENT connections have shown a significant influence on model performance. The interesting observation TITLE edges seem to play a leading role: eliminating it reduces the *F*1 by 5.47*%*. NEXT-SENT information also plays an essential role since removing it reduces *F*1 by 2.60*%*. Our proposed instance merging technique also has a significant contribution, without using it, *F*1 increases 3.22*%*. The shared-weight CNN on top-*k* paths demonstrated its good influence on the results by boosting *F*1 by 1.84*%*. Another experiment on using alternative methods for choosing top-*k* paths (by their repetitions frequencies instead of the shortest length) seems not suitable since it leads to a slight reduction in *F*1. As discussed above, the use of difference *w* for training and testing also brings a reduction of *F*1. Adding class weight and attention technique helps to improve *F*1 for 0.28*%* and 1.02*%*, respectively.

### Comparison to existing models

We compare the performance of our model against nine competitors. The first three models are capable of predicting intra sentence relations only, the next six models have the ability to extract inter sentence relations:

- Zhou et al. (2016) [41] proposed the hybridDNN model that consists of a feature-based model, a tree kernel-based model and a neural network model.
- Panyam et al. (2018) [42] used an enhanced dependency parse graph of a sentence with Approximate Subgraph Matching (ASM) kernel to classify CID relation.
- MASS [28] (stands for ‘Man for All Seasons’ model) is a large-scale neural relation classification architecture that has been applied on six benchmark datasets.
- UET-CAM system [23] is a Support vector machine (SVM) -based model. It uses multi-pass sieves for the coreference resolution to extract inter sentence relations.
- SVM-based model of Peng et al. (2016) [24] uses a rich feature set and rule-based output, enhanced by distant supervision learning.
- CNN+ME [25] is the hybrid model of the maximum entropy model for inter sentence relation classification and the CNN model for intra sentence relation classification.
- An LSTM-CNN model that learns document-level semantic representations by processing consecutive sentences as a sequence of sentences [20].
- Biaffine Relation Attention Network (BRAN) takes advantage of the state-of-the-art attention tool Transformer [17].
- The labeled edge graph convolutional neural network model on a document-level graph [18]. The graph is constructed using various inter- and intra sentence dependencies to capture local and non-local dependency information.

Table 6 summarizes the performance of our model and some comparative models. In which, the results of comparative models are reported both with and without using any additional enhancements.

**Table 6:** The performance of document subgraph-based model and some comparative models

Method/model		Precision	Recall	F1
*NOT having the ability to extract inter sentence relations*				
hybridDNN (Zhou et al., 2016 [41])	Syntactic features	62.15	47.28	53.70
	+ Context	62.39	47.47	53.92
	+ Position	62.86	47.47	54.09
ASM (Panyam et al., 2018 [42])	Dependency graph	49.00	67.40	56.80
MASS (Le et al., 2018 [28])	Multi channel CNN-LSTM	58.90	54.90	56.90
	+ Ensemble	56.80	57.90	57.30
	+ Post processing	52.80	71.10	60.60
*Having the ability to extract inter sentence relations*				
UET-CAM (Le et al., 2016 [23])	SVM + coreference	53.41	49.41	51.60
	+ Data	57.63	60.23	58.90
SVM (Peng et al., 2016 [24])	SVM + Rich feature set	64.24	52.06	57.51
	+ Data	**65.59**	56.94	61.01
CNN+ME (Gu et al., 2017 [25])	Hybrid model	60.90	59.50	60.20
	+ Post-processing	55.70	68.10	61.30
LSTM-CNN (Zheng et al., 2018 [20])	Sequence of sentences	24.00	52.00	32.80
	+ Entity replacing	54.30	65.90	59.50
BRAN (Verga et al., 2018 [17])	CNN + abstract attention	55.60	70.80	62.10
	+ Data	64.00	69.20	66.20
	+ Ensemble	65.40	71.80	68.40
Graph CNN (Sahu et al., 2019 [18])	Document-level Graph	52.80	66.00	58.60
Our results	Document subgraph	60.13	65.89	62.88
	+ Data	62.95	**75.16**	68.52
	+ Ensemble	64.79	74.05	**69.11**

Results are reported in %

Highest result in each column is highlighted in bold

Our model yields very competitive results when compared to other state-of-the-art models that have taken into account the inter sentence relationships. Compare to the original model without any additional enhancements, our model gives the best results with 62.88*%*.

Applying distant supervision learning and ensemble technique, our model still achieves the best result among competing models. The distant data helps to improves our *F*1 by 5.64*%* with the best hyper-parameter settings (this data also helped to boost the *F*1 by 5.9*%* in Peng et al. (2016) [24] and 4.1*%* in BRAN (Verga et al., 2018) [17]) The ensemble technique helps BRAN to boost the *F*1 for 2.2*%* whist it only helps our model for 0.6*%* more.

We also show the detailed results for intra- and inter sentence relation extraction in Table 7. In which, we exclude all inter sentence relations when evaluating intra sentence relation extraction results and vice versa.

**Table 7:** The detailed results of the document subgraph-based model

	Precision	Recall	F1
Full result	64.79	74.05	69.11
intra sentence relation result ^*†*^	72.91	85.73	78.80
inter sentence relation result ^*‡*^	46.12	47.28	46.69

Results are reported in %

Only evaluated on ^*†*^Intra- or ^*‡*^inter sentence relations

### Error analysis

We studied model outputs to analyze system errors and improvements as shown in Table 8. For further analysis, we use the output of RbSP- an advanced intra sentence relation extraction model [30]- for comparison, its results are shown in column *‘Comparative model’*. The full versions of the abstracts that used in Table 8 are given in Additional file 2: Appendix B.

**Table 8:** Examples of errors on the BC5 CDR test set

#	PMID	Chemical-Disease	Golden label	RbSP ^*†*^	SGM ^*‡*^	Type	Effect	Error type
1	2131034	D003561–D020258	CID	NONE	CID	Intra	**Better**	FN → TP
2	18801087	D000638–D009369	NONE	CID	NONE	Intra	**Better**	FP → TN
3	44072	C024986–D001145	CID	CID	NONE	Intra	*Worse*	TP → FN
4	15265979	D005947–D006529	NONE	NONE	CID	Intra	*Worse*	TN → FP
5	1655018	D000305–D006528	CID	NONE	NONE	Intra	−	FN
6	35781	D010423–D002375	NONE	CID	CID	Intra	−	FP
7	7644931	D017239–D018771	CID	−	CID	Inter	**Better**	FN → TP
8	10327032	D005472–D008107	NONE	−	CID	Inter	*Worse*	TN → FP
9	2710809	D001712–D003680	CID	−	−	Inter	−	FN
10	11745287	D016190–D015431	CID	−	NONE	Inter	−	FN
11	10087562	D004280–D008133	NONE	CID	CID	Intra	**Worse**^**∗**^	FN
12	24464946	D015251–D006331	NONE	−	CID	Inter	**Worse**^**∗**^	TN → FP

^*†*^The re-implemented intra sentence RbSP model (Can et al. [30]) - without subgraph model in Table 5

^*‡*^subgraph model’s prediction

*Errors due to the imperfect annotation

*CID* Chemical-induced disease, *NONE* Unrelated, ‘ −’: Cannot generate path, *TP* True Positive, *TN* True Negative, *FP* False Positive, *FN* False Negative

Cases where the SBM model gives correct results are highlighted in bold

The former part (Examples # 1−6) shows the effect of the graph-based model on intra sentence relations. It helps find some more intra sentence (Example # 1−2) relations since graph-based representation enriches many useful patterns for training. However, it also causes new noises (Example # 3−4), i.e., some examples are properly correctly labeled by the comparative model, but wrongly by the graph-based model. Example # 5−6 are errors that are not improved.

The latter part (examples # 7−10) focuses on the inter-relation extraction, these relations occupy about 30% of the instances in BC5 CDR corpus and cannot be extracted by the intra sentence model. Example #7 provides an improvement, as the graph model extracts the inter sentence relation correctly. In the case of producing false-positive results (Example #8), the graph-based model is penalized since turning a true negative into a false positive. Moreover, the graph model still misses many cases (Examples # 9−10).

These errors can be attributed to the limitations of our model, including *(a)* Many errors seem attributable to the parser. Example #9 is the case that we cannot generate any dependency path between two participated entities. The comprehensive analysis shows that our document subgraph representation with *w*=2 covers only ∼ 93*%* of total instances in test data (98% intra sentence relations and 87% inter sentence relations), in the remaining cases, we cannot generate any path between two entities. *(b)* The information in the path may still be insufficient or redundant to make the correct prediction. *(c)* The graph-based representation brings many noises. New virtual edge also brings confusion, and instance merging with top-*k* path choosing may lead to the missing of the useful paths. *(d)* The overfitting problem (leading to wrong prediction – *FP*) and *(e)* limited generalisation power in predicting new relations (*FN*).

Finally, we found some errors caused by the imperfect gold annotation (gold missing relation or gold false relation). Example #11 shows the case that our model finds a correct relation while gold standard annotation does not include. Another annotation errors (Example #12) come from the hierarchy manner. BC5 CDR corpus only annotates relations between the most specific entities, i.e., excludes the relations that involve entities that are more general than other entities already participated in the CID relation of each abstract [26].

## Discussion

In this work, we present a novel representation for a sequence of adjacent sentences in a document (namely document sub-graph). The graph is constructed using various types of information to capture local and non-local features. Knowledge-based information is also used to expropriate the manual realistic information to the model.

We also propose an instance merging mechanism and using a set of multiple paths for representing the relationship between entities pair. Our proposed model outperforms all comparative models in experiments on BC5 CDR corpus without using external knowledge resources and additional enhancements. Comparing the full model performance, our model still achieves comparable results when compared with the current state-of-the-art model (Verga’s BRAN model) [17].

When compared with the related work, the highlight of our proposed model is the use of document graphs with different train-test window sizes. To the best of our knowledge, most other studies approach in the direction of seeking relationships in one or several consecutive sentences [20, 24, 25, 28, 42]. Our model solves the problem of extracting relations in the whole document. This idea is similar to the study of Verga et al. [17], but they are in the direction of using the attention mechanism to find important information in the text. Instead, we build extract the information on the graph in a linguistic-based manner.

From the perspective of model usage in real-world applications, while graph building and model training are time consuming, they can be done offline. New data processing time is not fast enough to process big data but can be used to extract relations from small and medium datasets in reasonable time. Another problem when applying the model is processing full text. Through research and data survey, the abstract contains the basic information of the article. Basically, it is necessary to investigate more closely because the characteristics of full text and abstract are quite different. For example, with full text processing, window size of 5 may not be enough, two related entities may be very far apart. Extracting the relationship in full text will need some extra processing steps. We leave these problems for the future work.

We also investigated the results in detail to figure out our limitations for future improvements.

- Firstly, coreference and discourse resolutions should be analyzed carefully to find a suitable and more effective approach for application.
- Secondly, the valuable information coming from knowledge bases needs to be used more reasonably instead of being integrated directly into graphs.
- Thirdly, our model’s results resolutely depend on the performance of the dependency parser. This problem leads to the limitation that we must deal with many cascade errors from the processing step. We are planning to use another parser, which is specially built for the biomedical domain.
- Lastly, the ensemble mechanism should be improved to have higher results. However, run the graph-based models for many times is quite a time-consuming work; this approach needs an adaptation to be more suitable for the graph-based model.

## Conclusions

In this paper, we present a novel representation for a sequence of consecutive sentences in a document (namely document subgraph). The graph is constructed using various types of information to capture local and non-local features. We also propose an instance merging mechanism and use a set of multiple paths for representing the relationship between entity pairs. To explore the information in the document subgraph, we construct a deep neural architecture based on a shared-weight convolutional neural network.

The interesting analysis is that not all the types of new edges in the graph are useful for inter sentence rela- tion extraction. Only connections of title-sentences and between consecutive sentences are useful. In addition, all components and techniques that we applied in the proposed model show their contributions to the performance at a different level.

In experiments on BioCreative V CDR corpus, without using any external knowledge resources and additional enhancements, our proposed model outperforms all comparative models. We also investigated the results in detail to figure out our limitations for future improvement. The experimental results and error analysis help us to prioritize the future work.

## Supplementary Information

**Additional file 1** Appendix A. Example of a document subgraph.

**Additional file 2** Appendix B. The full versions of the abstracts that used in Table 8. Examples of errors on the BC5 CDR test set.

## Data Availability

The model implementation source code and sample parsed data will be available at https://github.com/catcd/subgraph-4re. Full parsed data is not public on github due to file size limit and is available from the corresponding author on reasonable request. We use the CTD-Pfizer dataset [34] for distant supervision learning. The BC5 CDR corpus evaluated in this study is provided by the BioCreative V task 3 CDR committee [26].

## Abbreviations

BC5: Biocreative V
BRANs: Biaffine Relation Attention Networks
CDR: Chemical-Disease Relation
CID: Chemical-Induced Disease
CNN: Convolutional Neural Network
CTD: Comparative Toxicogenomics Database
LSTM: Long Short-Term Memory network
LUT: Lookup Table
ME: Maximum Entropy
NLP: Natural Language Processing
POS tag: Part-of-speech tag
PubMed: a free search engine accessing primarily the MEDLINE database (a bibliographic database of life sciences and biomedical information)
RbSP: Richer-but-Smarter Shortest Dependency Path
RE: Relation Extraction
ReLU: Rectified Linear Unit
SDP: Shortest Dependency Path
SGM: subgraph model
SVM: Support Vector Machine
swCNN: shared-weight Convolutional Neural Network

## Footnotes

1 http://www.ctdbase.org

2 Information about this TensorFlow version is at https://www.tensorflow.org/versions/r1.15/api_docs/python/tf

3 spaCy: Industrial-Strength Natural Language Processing in Python: https://spacy.io
