# Supplementary material for: Exploiting document graphs for inter sentence relation extraction
Source: J Biomed Semantics. 2022 Jun 3;13:15. doi: 10.1186/s13326-022-00267-3 (PMC9166375; doi:10.1186/s13326-022-00267-3)
Supplement: Supplementary file 1 — Additional file 1 Appendix A. Example of a document subgraph. [file 13326_2022_267_MOESM1_ESM.pdf]

Appendix A.

Example of a document sub-graph

ORIGINAL ABSTRACT: PMID 16740173
(In which, example sentences are highlighted in bold (#6-7-8))

<TITLE>
Case report: acute unintentional carbachol intoxication.
<ABSTRACT>
INTRODUCTION: Intoxications with carbachol, a muscarinic cholinergic receptor agonist are rare. We report an interesting case investigating a (near) fatal poisoning. METHODS: The son of an 84-year-old male discovered a newspaper report stating clinical success with plant extracts in Alzheimer's disease. The mode of action was said to be comparable to that of the synthetic compound 'carbamylcholin'; that is, carbachol. He bought 25 g of carbachol as pure substance in a pharmacy, and the father was administered 400 to 500 mg. **Carbachol concentrations in serum and urine on day 1 and 2 of hospital admission were analysed by HPLC-mass spectrometry. RESULTS: Minutes after oral administration, the patient developed nausea, sweating and hypotension, and finally collapsed. Bradycardia, cholinergic symptoms and asystole occurred.** Initial cardiopulmonary resuscitation and immediate treatment with adrenaline (epinephrine), atropine and furosemide was successful. On hospital admission, blood pressure of the intubated, bradyarrhythmic patient was 100/65 mmHg. Further signs were hyperhidrosis, hypersalivation, bronchorrhoea, and severe miosis; the electrocardiographic finding was atrio-ventricular dissociation. High doses of atropine (up to 50 mg per 24 hours), adrenalineand dopamine were necessary. The patient was extubated 1 week later. However, increased dyspnoea and bronchospasmnecessitated reintubation. Respiratory insufficiency was further worsened by Proteus mirabilis infection and severe bronchoconstriction. One week later, the patient was again extubated and 3 days later was transferred to a peripheral ward. On the next day he died, probably as a result of heart failure. Serum samples from the first and second days contained 3.6 and 1.9 mg/l carbachol, respectively. The corresponding urine concentrations amounted to 374 and 554 mg/l. CONCLUSION: This case started with a media report in a popular newspaper, initiated by published, peer-reviewed research on herbals, and involved human failure in a case history, medical examination and clinical treatment. For the first time, an analytical method for the determination of carbachol in plasma and urine has been developed. The analysed carbachol concentration exceeded the supposed serum level resulting from a therapeutic dose by a factor of 130 to 260. Especially in old patients, intensivists should consider intoxications (with cholinergics) as a cause of acute cardiovascular failure.

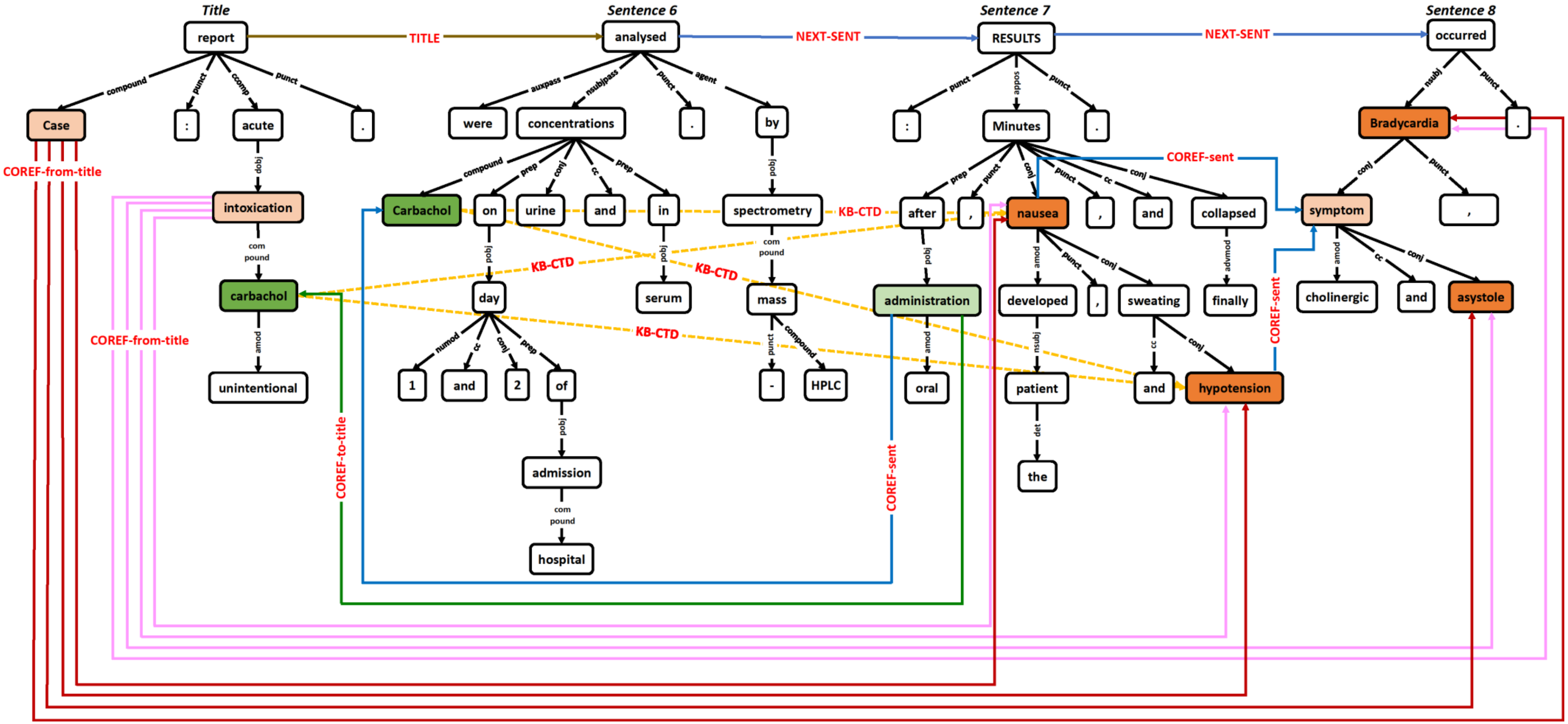
Title
Sentence 6
Sentence 7
Sentence 8
report
TITLE
analysed
NEXT-SENT
RESULTS
NEXT-SENT
occurred
compound
punct
ccomp
punct
Case
:
acute
.
COREF-from-title
dobj
intoxication
com
pound
carbachol
amod
unintentional
COREF-from-title
auxpass
nsubjpass
punct
agent
were
concentrations
.
by
compound
prep
conj
cc
prep
Carbachol
on
urine
and
in
pobj
spectrometry
KB-CTD
pobj
pobj
KB-CTD
KB-CTD
day
serum
com
pound
mass
nummod
cc
conj
prep
1
and
2
of
punct
compound
-
HPLC
KB-CTD
pobj
admission
com
pound
hospital
COREF-to-title
punct
appos
punct
:
Minutes
.
COREF-sent
prep
punct
conj
punct
cc
conj
after
,
nausea
,
and
collapsed
pobj
administration
amod
oral
amod
punct
conj
developed
,
sweating
advmod
finally
nsubj
patient
det
the
cc
conj
and
hypotension
COREF-sent
COREF-sent
nsubj
punct
Bradycardia
.
conj
punct
symptom
,
amod
cc
conj
cholinergic
and
asystole
