# Supplementary material for: Exploiting document graphs for inter sentence relation extraction
Source: J Biomed Semantics. 2022 Jun 3;13:15. doi: 10.1186/s13326-022-00267-3 (PMC9166375; doi:10.1186/s13326-022-00267-3)
Supplement: Supplementary file 2 — Additional file 2 Appendix B. The full versions of the abstracts that used in Table 8. Examples of errors on the BC5 CDR test set. [file 13326_2022_267_MOESM2_ESM.pdf]

Appendix B

The full versions of the abstracts that used in Table 8. Examples of errors on the BC5 CDR test set.

Example 1.

PMID 2131034: D003561-D020258

TITLE:
Severe polyneuropathy and motor loss after intrathecal thiotepa combination chemotherapy: description of two cases.
ABSTRACT:
Two cases of severe delayed neurologic toxicity related to the administration of intrathecal (IT) combination chemotherapy including thiotepa (TSPA) are presented. Both cases developed axonal neuropathy with motor predominance in the lower extremities 1 and 6 months after IT chemotherapy was administered. Neurologic toxicities have been described with IT-methotrexate, IT-cytosine arabinoside and IT-TSPA. To our knowledge, however, axonal neuropathy following administration of these three agents has not been previously described. In spite of the fact that TSPA is a useful IT agent, its combination with MTX, ara-C and radiotherapy could cause severe neurotoxicity. This unexpected complication indicates the need for further toxicology research on IT-TSPA.

Example 2.

PMID 18801087: D000638-D009369

TITLE:
Amiodarone-related pulmonary mass and unique membranous glomerulonephritis in a patient with valvular heart disease: Diagnostic pitfall and new findings.
ABSTRACT:
Amiodarone is an anti-arrhythmic drug for life-threatening tachycardia, but various adverse effects have been reported. Reported herein is an autopsy case of valvular heart disease, in a patient who developed a lung mass (1.5 cm in diameter) and proteinuria (2.76 g/day) after treatment with amiodarone for a long time. The lung mass was highly suspected to be lung cancer on CT and positron emission tomography, but histologically the lesion was composed of lymphoplasmacytic infiltrates in alveolar walls and intra-alveolar accumulation of foamy macrophages containing characteristic myelinoid bodies, indicating that it was an amiodarone-related lesion. In addition, the lung tissue had unevenly distributed hemosiderin deposition, and abnormally tortuous capillaries were seen in the mass and in heavily hemosiderotic lung portions outside the mass. In the kidneys, glomeruli had membrane spikes, prominent swelling of podocytes and subepithelial deposits, which were sometimes large and hump-like. Autoimmune diseases, viral hepatitis, malignant neoplasms or other diseases with a known relationship to membranous glomerulonephritis were not found. The present case highlights the possibility that differential diagnosis between an amiodarone-related pulmonary lesion and a neoplasm can be very difficult radiologically, and suggests that membranous glomerulonephritis might be another possible complication of amiodarone treatment.

Example 3.

PMID 44072: C024986-D001145

TITLE:
On the antiarrhythmic activity of one N-substituted piperazine derivative of trans-2-amino-3-hydroxy-1, 2, 3, 4-tetrahydroanaphthalene.
ABSTRACT:
The antiarrhythmic activity of the compound N-(trans-3-hydroxy-1,2,3,4-tetrahydro-2-naphthyl)-N-(3-oxo-3-phenyl-2-methylpropyl)-piperazine hydrochloride, referred to as P11, is studied on anaesthesized cats and Wistar albino rats, as well as on non-anaesthesized rabbits. Four types of experimental arrhythmia are used--with $BaCl_2$, with

chloroform-adrenaline, with strophantine G and with aconitine. The compound P11 is introduced in doses of 0.25 and 0.50 mg/kg intravenously and 10 mg/kg orally. The compound manifests antiarrhythmic activity in all models of experimental arrhythmia used, causing greatest inhibition on the arrhythmia induced by chloroform-adrenaline (in 90 per cent) and with BaCl2 (in 84 per cent). The results obtained are associated with the beta-adrenoblocking and with the membrane-stabilizing action of the compound.

Example 4.

PMID 15265979: D005947-D006529

TITLE:
Disruption of hepatic lipid homeostasis in mice after amiodarone treatment is associated with peroxisome proliferator-activated receptor-alpha target gene activation.
ABSTRACT:
Amiodarone, an efficacious and widely used antiarrhythmic agent, has been reported to cause hepatotoxicity in some patients. To gain insight into the mechanism of this unwanted effect, mice were administered various doses of amiodarone and examined for changes in hepatic histology and gene regulation. Amiodarone induced hepatomegaly, hepatocyte microvesicular lipid accumulation, and a significant decrease in serum triglycerides and glucose. Northern blot analysis of hepatic RNA revealed a dose-dependent increase in the expression of a number of genes critical for fatty acid oxidation, lipoprotein assembly, and lipid transport. Many of these genes are regulated by the peroxisome proliferator-activated receptor-alpha (PPARalpha), a ligand-activated nuclear hormone receptor transcription factor. The absence of induction of these genes as well as hepatomegaly in PPARalpha knockout [PPARalpha-/-] mice indicated that the effects of amiodarone were dependent upon the presence of a functional PPARalpha gene. Compared to wild-type mice, treatment of PPARalpha-/- mice with amiodarone resulted in an increased rate and extent of total body weight loss. The inability of amiodarone to directly activate either human or mouse PPARalpha transiently expressed in human HepG2 hepatoma cells indicates that the effects of amiodarone on the function of this receptor were indirect. Based upon these results, we conclude that amiodarone disrupts hepatic lipid homeostasis and that the increased expression of PPARalpha target genes is secondary to this toxic effect. These results provide important new mechanistic information regarding the hepatotoxic effects of amiodarone and indicate that PPARalpha protects against amiodarone-induced hepatotoxicity.

Example 5.

PMID 1655018: D000305--D006528

TITLE:
Hepatocellular carcinoma in Fanconi's anemia treated with androgen and corticosteroid.
ABSTRACT:
The case of an 11-year-old boy is reported who was known to have Fanconi's anemia for 3 years and was treated with androgens, corticosteroids and transfusions. Two weeks before his death he was readmitted because of aplastic crisis with septicemia and marked abnormalities in liver function and died of hemorrhagic bronchopneumonia. At autopsy peliosis and multiple hepatic tumors were found which histologically proved to be well-differentiated hepatocellular carcinoma. This case contributes to the previous observations that non-metastasizing hepatic neoplasms and peliosis can develop in patientswith androgen- and corticosteroid-treated Fanconi's anemia.

Example 6.

PMID 35781: D010423--D002375

TITLE:
Central action of narcotic analgesics. Part IV. Noradrenergic influences on the activity of analgesics in rats.
ABSTRACT:

The effect of clonidine, naphazoline and xylometazoline on analgesia induced by morphine, codeine, fentanyl and pentazocine, and on cataleptic effect of morphine, codine and fentanyl was studied in rats. The biochemical assays on the influence of four analgesics on the brain concentration and turnover of noradrenaline (NA) were also performed. It was found that three drugs stimulating central NA receptors failed to affect the analgesic ED50 of all antinociceptive agents and they enhanced catalepsy induced by morphine and fentanyl. Codeine catalepsy was increased by clonidine and decreased by naphazoline and xylometazoline. The brain concentration of NA was not changed by morphine and fentanyl, but one of the doses of codeine (45 mg/kg) slightly enhanced it. Pentazocine dose-dependently decreased the brain level of NA. The rate of NA turnover was not altered by analgesics except for the higher dose of fentanyl (0.2 mg/kg) following which the disappearance of NA from the brain was diminished. The results are discussed in the light of various and non-uniform data from the literature. It is suggested that in rats the brain NA plays a less important function than the other monoamines in the behavioural activity of potent analgesics.

Example 7.

PMID 7644931: D017239--D018771

TITLE:
Paclitaxel 3-hour infusion given alone and combined with carboplatin: preliminary results of dose-escalation trials.
ABSTRACT:
Paclitaxel (Taxol; Bristol-Myers Squibb Company, Princeton, NJ) by 3-hour infusion was combined with carboplatin in a phase I/II study directed to patients with non-small cell lung cancer. Carboplatin was given at a fixed target area under the concentration-time curve of 6.0 by the Calvert formula, whereas paclitaxel was escalated in patient cohorts from 150 mg/m2 (dose level I) to 175, 200, 225, and 250 mg/m2. The 225 mg/m2 level was expanded for the phase II study since the highest level achieved (250 mg/m2) required modification because of nonhematologic toxicities (arthralgia and sensory neuropathy). Therapeutic effects were noted at all dose levels, with objective responses in 17 (two complete and 15 partial regressions) of 41 previously untreated patients. Toxicities were compared with a cohort of patients in a phase I trial of paclitaxel alone at identical dose levels. Carboplatin did not appear to add to the hematologic toxicities observed, and the paclitaxel/carboplatin combination could be dosed every 3 weeks.

Example 8.

PMID 10327032: D005472-D008107

TITLE:
Risk of transient hyperammonemic encephalopathy in cancer patients who received continuous infusion of 5-fluorouracil with the complication of dehydration *&* infection.
ABSTRACT:
From 1986 to 1998, 29 cancer patients who had 32 episodes of transient hyperammonemic encephalopathy related to continuous infusion of 5-fluorouracil (5-FU) were identified. None of the patients had decompensated liver disease. Onset of hyperammonemic encephalopathy varied from 0.5 to 5 days (mean: 2.6 +/- 1.3 days) after the initiation of chemotherapy. Plasma ammonium level ranged from 248 to 2387 microg% (mean: 626 +/- 431 microg%). Among the 32 episodes, 26 (81%) had various degrees of azotemia, 18 (56%) occurred during bacterial infections *&* 14 (44%) without infection occurred during periods of dehydration. Higher plasma ammonium levels *&* more rapid onset of hyperammonemia were seen in 18 patients with bacterial infections (p=0.003 *&* 0.0006, respectively) *&* in nine patients receiving high daily doses (2600 or 1800 mg/m2) of 5-FU (p=0.0001 *&* < 0.0001, respectively). In 25 out of 32 episodes (78%), plasma ammonium levels *&* mental status returned to normal within 2 days after adequate management. In conclusion, hyperammonemic encephalopathy can occur in patients receiving continuous infusion of 5-FU. Azotemia, body fluid insufficiency *&* bacterial infections were frequently found in these patients. It is therefore important to recognize this condition in patientsreceiving continuous infusion of 5-FU.

Example 9.

PMID 2710809: D001712--D003680

TITLE:
Bradycardia due to biperiden.
ABSTRACT:
In a 38-year-old male patient suffering from a severe postzosteric trigeminal neuralgia, intravenous application of 10 mg biperiden lactate led to a long-lasting paradoxical reaction characterized by considerable bradycardia, dysarthria, and dysphagia. The heart rate was back to normal within 12 hours upon administration of orciprenaline under cardiac monitoring in an intensive care unit. Bradycardia induced by biperiden is attributed to the speed of injection and to a dose-related dual effect of atropine-like drugs on muscarine receptors.

Example 10.

PMID 11745287: D016190-D015431

TITLE:
Phase II study of carboplatin and liposomal doxorubicin in patients with recurrent squamous cell carcinoma of the cervix.
ABSTRACT:
BACKGROUND: The activity of the combination of carboplatin and liposomal doxorubicin was tested in a Phase II study of patientswith recurrent cervical carcinoma. METHODS: The combination of carboplatin (area under the concentration curve [AUC], 5) and liposomal doxorubicin (Doxil; starting dose, 40 mg/m(2)) was administered intravenously every 28 days to 37 patients with recurrent squamous cell cervical carcinoma to determine antitumor activity and toxicity profile. RESULTS: Twenty-nine patients were assessable for response, and 35 patients were assessable for toxicity. The overall response rate was 38%, the median time to response was 10 weeks, the median duration of response was 26 weeks, and the median survival was 37 weeks. The main toxic effect was myelosuppression, with Grade 3 and 4 neutropenia in 16 patients, anemia in 12 patients, thrombocytopenia in 11 patients, and neutropenic fever in 3 patients. Four patients had five infusion-related reactions during the infusion of liposomal doxorubicin, leading to treatment discontinuation in three patients. Grade > or = 2 nonhematologic toxicity included nausea in 17 patients, emesis in 14 patients, fatigue in 9 patients, mucositis and/or stomatitis in 8 patients, constipation in 6 patients, weight loss in 5 patients, hand-foot syndrome in 2 patients, and skin reactions in 3 patients. CONCLUSIONS: The combination of carboplatin and liposomal doxorubicin has modest activity in patients with recurrent cervical carcinoma.

Example 11.

PMID 10087562: D004280-D008133

TITLE:
Torsade de pointes ventricular tachycardia during low dose intermittent dobutamine treatment in a patient with dilated cardiomyopathy and congestive heart failure.
ABSTRACT:
The authors describe the case of a 56-year-old woman with chronic, severe heart failure secondary to dilated cardiomyopathy and absence of significant ventricular arrhythmias who developed QT prolongation and torsade de pointes ventricular tachycardia during one cycle of intermittent low dose (2.5 mcg/kg per min) dobutamine. This report of torsade de pointes ventricular tachycardia during intermittent dobutamine supports the hypothesis that unpredictable fatal arrhythmias may occur even with low doses and in patients with no history of significant rhythm disturbances. The mechanisms of proarrhythmic effects of Dubutamine are discussed.

Example 12.

PMID 24464946: D015251--D006331

TITLE:
Two-dimensional speckle tracking echocardiography combined with high-sensitive cardiac troponin T in early detection and prediction of cardiotoxicity during epirubicine-based chemotherapy.

ABSTRACT:
AIMS: To investigate whether alterations of myocardial strain and high-sensitive cardiac troponin T (cTnT) could predict future cardiac dysfunction in patients after epirubicin exposure. METHODS: Seventy-five patients with non-Hodgkin lymphoma treated with epirubicin were studied. Blood collection and echocardiography were performed at baseline, 1 day after the third cycle, and 1 day after completion of chemotherapy. Patients were studied using echocardiography during follow-up. Global longitudinal (GLS), circumferential (GCS), and radial strain (GRS) were calculated using speckle tracking echocardiography. Left ventricular ejection fraction was analysed by real-time 3D echocardiography. Cardiotoxicity was defined as a reduction of the LVEF of >=5% to <55% with symptoms of **heart failure** or an asymptomatic reduction of the LVEF of >=10% to <55%. RESULTS: Fourteen patients (18.67%) developed cardiotoxicity after treatment. GLS (-18.48 +- 1.72% vs. -15.96 +- 1.6%), GCS (-20.93 +- 2.86% vs. -19.20 +- 3.21%), and GRS (39.23 +- 6.44% vs. 34.98 +- 6.2%) were markedly reduced and cTnT was elevated from 0.0010 +- 0.0020 to 0.0073 +- 0.0038 ng/mL (P all < 0.01) at the completion of chemotherapy compared with baseline values. A >15.9% decrease in GLS [sensitivity, 86%; specificity, 75%; area under the curve (AUC) = 0.815; P = 0.001] and a >0.004 ng/mL elevation in cTnT (sensitivity, 79%; specificity, 64%; AUC = 0.757; P = 0.005) from baseline to the third cycle of chemotherapy predicted later cardiotoxicity. The decrease in GLS remained the only independent predictor of cardiotoxicity (P = 0.000). CONCLUSIONS: GLS combined with cTnT may provide a reliable and non-invasive method to predict cardiac dysfunction in patients receiving anthracycline-based chemotherapy.
